# Using neutral cline decay to estimate contemporary dispersal: a generic tool and its application to a major crop pathogen

**DOI:** 10.1111/ele.12090

**Published:** 2014-07-22

**Authors:** A Rieux, T Lenormand, J Carlier, L de Lapeyre de Bellaire, V Ravigné

**Affiliations:** 1 CIRAD, UMR BGPICampus international de Baillarguet, TA A-54K, F-34398, Montpellier Cedex 5, France; 2 CEFE-CNRS, UMR 51751919 route de Mende, F-34293, Montpellier Cedex 5, France; 3 CIRAD, Persyst, UPR Syst. Banan. AnanasTA B-26/PS4, Blvd. de la Lironde, 34398, Montpellier Cedex 5, France

**Keywords:** Dispersal ecology, emergent disease, gene flow estimation, genetic clines, landscape genetics, *Mycosphaerella fijiensis*, neutral markers, non-equilibrium populations, secondary contact

## Abstract

Dispersal is a key parameter of adaptation, invasion and persistence. Yet standard population genetics inference methods hardly distinguish it from drift and many species cannot be studied by direct mark-recapture methods. Here, we introduce a method using rates of change in cline shapes for neutral markers to estimate contemporary dispersal. We apply it to the devastating banana pest *Mycosphaerella fijiensis*, a wind-dispersed fungus for which a secondary contact zone had previously been detected using landscape genetics tools. By tracking the spatio-temporal frequency change of 15 microsatellite markers, we find that σ, the standard deviation of parent–offspring dispersal distances, is 1.2 km/generation^1/2^. The analysis is further shown robust to a large range of dispersal kernels. We conclude that combining landscape genetics approaches to detect breaks in allelic frequencies with analyses of changes in neutral genetic clines offers a powerful way to obtain ecologically relevant estimates of dispersal in many species.

## Introduction

Virtually every ecological and evolutionary process is influenced by dispersal. From an ecological perspective, dispersal influences population dynamics and persistence, species distribution and abundance, and community structure (Dieckmann *et al*. 1999). It is particularly critical for biological invasions (Lockwood *et al*. 2005). From an evolutionary perspective, dispersal determines the level of gene flow between populations and therefore affects processes such as local adaptation and species range (Lenormand 2002), speciation (e.g. reviewed in Barton 2001), and the evolution of life history traits (examples in Ronce 2007). For pathogens, dispersal results in disease spread (Brown & Hovmøller 2002) and might condition their evolutionary potential (McDonald & Linde 2002).

Despite its importance, estimating dispersal in natural populations remains challenging (Rousset 2001). There is a variety of techniques ranging from direct observation to indirect (genetic-based) measures of dispersal-related processes. Each method has drawbacks and advantages (Rousset 2001; Broquet & Petit 2009). For instance, only genetic-based inference methods are able to quantify effective gene (rather than individual) movements but contrary to direct methods, which rely on few assumptions, they critically depend on an appropriate modelling of a combination of underlying processes. This occurs in models inferring dispersal from patterns of genetic variation expected at drift–dispersal equilibrium (e.g. isolation by distance, Rousset 1997) or selection–dispersal equilibrium (e.g. tension zones, reviewed in Barton & Hewitt 1985). Models at drift–dispersal equilibrium present the specific drawback that they require independent information on effective population densities to infer dispersal rates (Slatkin & Barton 1989; Rousset 1997). Besides, the provided estimates reflect both historical and contemporary dispersal events, hampering comparisons with direct measurements. Parentage assignment analyses have been developed to infer contemporary dispersal (reviewed in Broquet & Petit 2009) but they rely on an extensive sampling of both parents and their progeny, which is infeasible in many species. Alternatively, methods at selection–dispersal equilibrium consider clines maintained, either by selection against hybrids between two taxa (tension zones) or by different selection pressures in geographically adjacent areas. In such situations, provided multilocus clines are observed, measures of linkage disequilibria between loci allow estimating short-term dispersal parameters (Barton 1982; Barton & Hewitt 1985 for tension zones, Lenormand *et al*. 1998 for local adaptation). There also have been attempts to exploit non-equilibrium situations such as (1) cline temporal variation (e.g. the speed of a wave of advance of a Wolbachia infection, Turelli & Hoffmann 1991) or (2) cline shape rate of change when selection or migration varies (Lenormand & Raymond 2000; Saccheri *et al*. 2008). Despite its potential for estimating dispersal, genetic cline theory (whether or not at equilibrium) has seldom been used in comparison to other methods. One possible explanation could be that situations near dispersal-selection equilibrium except hybrid zones are difficult to identify in natural populations. The aim of this article was to illustrate how contact zones in a purely neutral situation can be useful to estimate dispersal in various species.

Genetic clines, that are spatial gradients in gene frequencies, may be observed for neutral polymorphic markers when divergent populations meet after having been separated by some climatic or geological change or following introduction into a new region (secondary contact). Such neutral clines are not stable through time (Endler 1977). Provided that there is no selection (either antagonistic between the areas or against hybrid genotypes), the initial step in allele frequencies should gradually dissolve, leaving only a smooth vanishing cline. Under conditions detailed below, Endler (1977) found a relationship between neutral cline width, migration intensity and the time since contact. Surprisingly, this theory has not been used yet to estimate dispersal in natural populations, probably for several reasons. First, detecting secondary contact zones may be difficult. Accurate localisation of contact zones requires two-dimensional sampling strategies and spatially explicit methods of analysis. With the recent emergence of landscape genetics, such methods now exist (reviewed in Guillot *et al*. 2009). Second, time since historical contact is rarely known. Third, Endler∼s formula applies in absence of selection or drift. Its application therefore requires diagnosing whether drift and selection can be neglected in the studied system. Last, an important underlying assumption is that migration amounts to a diffusion process, which may not be true in biological systems where long-distance dispersal events occur.

In this article, we show how the rate of change of neutral cline width over a known number of generations allows estimating dispersal in natural populations in absence of information about contact time. We further show that, using a maximum likelihood framework, one may test whether clines evolve under the effect of migration only. We apply this method to the fungus *Mycosphaerella fijiensis*, a wind-dispersed plant pathogen causing black leaf streak disease (BLSD) of banana. BLSD is the most economically important disease of bananas (Jones 2000) and is among the most important crop diseases in the world (Pennisi 2010). A better knowledge of dispersal is particularly required to improve strategies of disease control and fungicide resistance management (Lenormand & Raymond 1998; de Lapeyre de Bellaire *et al*. 2010). *Mycosphaerella fijiensis* has recently spread worldwide from South-East Asia (Carlier 2000). It has been first mentioned in 1973 in Africa and in 1980 in Cameroon (Mourichon & Fullerton 1990). All previous attempts to measure its dispersal through indirect methods have failed, because its population structure reflects historical patterns of colonisation rather than current equilibrium between evolutionary forces (Halkett *et al*. 2010; Rieux *et al*. 2011). In a previous landscape genetics study in South-West Cameroon, Rieux *et al*. (2011) have detected a contact area between two expanding populations. Their contact time is unknown. In the following, we use spatially explicit genetic clustering to show that the genetic discontinuity previously detected is still present 2 years later. We find the best description of neutral clines around the contact area for 15 microsatellite loci. We use the spatio-temporal evolution of these clines to estimate dispersal in *M. fijiensis*. A maximum likelihood framework allows measuring estimation quality and the validity of the pure-migration hypothesis. Finally, using theoretical simulations, we assess the robustness of the analysis to deviations from the diffusion approximation. We discuss the validity of our results and the portability of the method to other species.

## Materials and methods

### Study area and sampling

**Figure 1:**
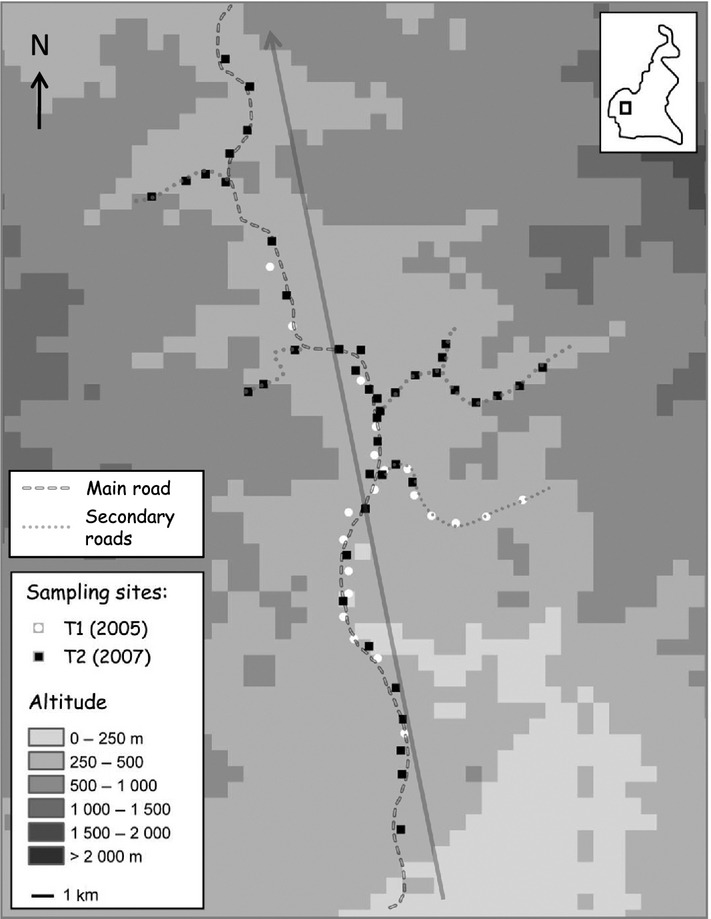
Sample site locations along the transect in South-West Cameroon. The arrow indicates the best-fit axis θ as inferred using the neutral cline approach.

*Mycosphaerella fijiensis* samples were collected in accessible areas along the roadside verges of a South-West Cameroonian agricultural landscape. At each sampling site, infected leaf fragments were collected from a few neighbour banana plants, and GPS coordinates were recorded. Two samplings were realised in November 2005 (T_1_, included in Rieux *et al*. (2011)) and February 2007 (T_2_) (Fig. 1, Appendix S1). We estimated that 15 pathogen generations of sexual reproduction have occurred between the two samplings (Jones 2000). Three to eight individuals were isolated per sampling site. At T_1_, 21 sites were sampled along a 32-km long transect. Mycelium cultures were initiated by single ascospores isolated from necrotic lesions grown on solid medium (Halkett *et al*. 2010). At T_2_, 44 sites were sampled along a 45-km long transect and thanks to new technical developments, the laborious pathogen isolation step was avoided by collecting young lesions directly from banana leaves (Robert *et al*. 2010).

### Genotyping

Total genomic DNA was either extracted from mycelial cultures (T_1_) or from young lesions (T_2_). Genotyping was carried out using 15 microsatellites loci (Neu *et al*. 1999; Zapater *et al*. 2008; Robert *et al*. 2010). Protocols and polymerase chain reaction conditions are detailed in Robert *et al*. (2010). Among the 15 markers, two are tri-nucleotide and four are di-nucleotide microsatellite loci already used in the 2011 study. The nine others are tetra-nucleotide markers developed between the two studies (Appendix S3). Amplified fragments were separated on a 16-capillary Sequencer (ABI Prism 3130XL (Applied Biosystems, Foster City, CA, USA) and analysed for length variation using GeneMapper® Software. In total, 144 individuals (T_1_) and 244 individuals (T_2_) were genotyped.

### Delineating the genetic discontinuity using spatial genetic clustering

Spatial population genetic structure was assessed using the clustering algorithms implemented in geneland v3.1.4 (Guillot *et al*. 2005) and tess v2.3.1 (Chen *et al*. 2007). Both algorithms distribute individual genotypes into *K* clusters by minimising Hardy–Weinberg disequilibrium and gametic phase disequilibrium within groups, taking into account spatial coordinates. In geneland, we used the independent model of allele frequencies. We ran tess using an admixture model defined according to the BYM model. In both analyses, we performed five independent runs with 10^6^ iterations (including 10% of burn-in period) allowing *K* to vary from 1 to 10 in geneland and from 2 to 10 in tess. *K* was inferred from the modal value of the run highest posterior probability in geneland and from the mean value of the run lowest deviance information criterion (DIC) in tess. For the best *K*, posterior individual membership probability to each genetic group or parental population was averaged across best runs∼ outputs. We used the mba.surf function of R software (R Core Team 2012) to map the distribution of genetic groups over each pixel of the spatial domain considered.

### Population structure

On the entire dataset and for both dates, gene diversity was assessed using expected heterozygosity (*H*_E_) and the number of alleles (*N*_A_) using fstat_2.9.3_ (Goudet 1995). We used the nonparametric Wilcoxon–Mann–Whitney *U*-test to detect differences in heterozygosity between dates. Linkage disequilibrium between all pairs of loci was tested using Fisher∼s exact tests implemented in genepop (Rousset 2008). The false discovery rate (FDR) procedure implemented in the R package qvalue (Storey 2002) allowed controlling for multiple testing. Genetic differentiation between clusters was estimated through *F*_*ST*_ using genepop. Likelihood ratio *G* test allowed estimating the significance of *F*_*ST*_ values.

### Description of neutral genetic clines

#### Cline fitting tool

To fit genetic clines, we used the software CFit7 (Gay *et al*. 2008, available at http://www.cefe.cnrs.fr/genetique-et-ecologie-evolutive/cfit), which we modified to account for 2D data. It allows fitting bi-allelic allele count data to geographical distance using various cline shapes. Several clines can be fitted to compare their slopes (concordance) and position (coincidence) by constraining shape parameters. Estimation is performed by maximum likelihood. Likelihood is computed considering that allelic counts in a site are drawn from a binomial distribution. It is maximised using a simulated annealing algorithm. Here, most genetic markers had only two alleles (Appendix S3). The others were converted to two-allele systems by assigning each allele to a cluster-specific compound allele according to its coordinates on the first axis of a multiple correspondence analysis (MCA) (Daguin *et al*. 2001). Because sampling sites were not strictly aligned, we used 2-dimensional clines.

#### Cline description

We first determined how to best describe the data by fitting three different 2D cline shapes to multilocus genetic data independently for T_1_ and T_2_ without any constraint on parameters. To do so, spatial coordinates (*X*, *Y*) were transformed into a single coordinate along the cline axis using:


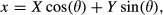
(1)

where θ is the angle between cline axis and the parallel (East–West line). We then fitted three functions of *x* to allelic count data: two Logit shapes consistent with the neutral hypothesis and a stepped cline expected in tension zones involving strong indirect selection across the genome (details and full equations in Appendix S4). Second, using the best shape, for each locus, we first determined whether a cline was present at T_1_ (non-zero slope) using likelihood ratio tests (LRT). Third, for each locus presenting a significant cline pattern at T_1_, we tested for a slope change between T_1_ and T_2_ using LRT again. Model choice was made at the 5% significance level using a chi-squared test at d.f. difference in freedom degrees between the compared models.

### Dispersal estimation

Cline width (*w*) is the ratio between the maximal difference in allele frequencies along the cline and maximal cline slope:


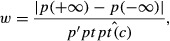
(2)

where *p*(.) is allele frequency, *p′*(.) its derivative with respect to geographical distance and 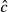 the estimated cline centre. Dispersal estimation was based on the multilocus cline width rate of change. Following a secondary contact, assuming no difference in density between the two groups, negligible drift and diffusion approximation for dispersal, cline width should decay at a rate only determined by the standard deviation of parent–offspring axial dispersal distances (σ) and the time since contact (*T* ) (Endler 1977; Gay *et al*. 2008):


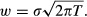
(3)

Cline width at T_2_ (*w*_2_) thus depends on cline width at T_1_ (*w*_1_) and dispersal (σ):


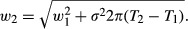
(4)

Because the contact zone may move with time, for example under asymmetric gene flow (Barton & Hewitt 1985), cline centres were also allowed to differ between T_1_ and T_2_ by a distance shift *δ*. As detailed in Appendix S6, we reparameterised eqn 4 using the scaled Logit shape and integrated this equation in a new version of CFit7 (available online) thus allowing a maximum likelihood estimation of dispersal (jointly with cline angle, cline centre and centre shift) from the full dataset.

Loci without any cline pattern at T_1_ were included in the likelihood computation assuming a constant allele frequency over all sites at T_1_. Likelihood was corrected for overdispersal of genetic data using the variance inflation factor (Lebreton *et al*. 1992), estimated from the residual deviance of the complete model divided by its residual degrees of freedom. Model selection was done using Akaike criterion. Support limits for σ and θ were estimated within 2 units around the maximum likelihood value.

### Influence of kernel on dispersal estimation

We investigated whether dispersal kernels deviating from diffusion could bias the estimation of σ. We simulated the evolution of genetic clines under different dispersal distributions with varying kurtosis and conducted the estimation procedure as above. We considered a circular one-dimensional space divided in *n* sites connected by migration. Migration was modelled with five mixtures of Binomial (Δ_0_ → Δ_4_), as used in previous theoretical studies about kernel-mediated biases in cline approaches, and 11 distributions (Δ_5_ → Δ_16_) belonging to three different kernel families commonly used to model dispersal kernels: Exponential power, 2Dt and Geometric (full equations in Appendix S9). For each kernel family, parameter values were chosen so that all distributions have the same variance (σ^2^), corresponding to the value inferred from the real data, but different kurtosis (γ ranging from −1 to ∼ 100, Fig. 4a). We then simulated the evolution of allele frequency at one bi-allelic neutral locus following a secondary contact under each of these dispersal kernels over 200 generations (Fig. 4b). Producing the next generation amounted to convolve the list of allelic frequencies at generation *t* with migration kernel so that at each site *i*, allele frequency at generation *t* + 1 is given by:


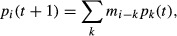
(5)

where *m* is the migration kernel. Dispersal was then estimated for all dispersal kernels and for each of them by varying first sampling time (T_1_) from 1 to 185 generations after secondary contact. All simulations were performed with Mathematica 7.0 (Wolfram Research, Inc., Champaign, IL, USA).

## Results

### Delineating the genetic discontinuity

In both datasets (T_1_ and T_2_), geneland and tess clearly detected two genetic groups (Appendix S2). In T_1_, applying an assignment threshold of 0.80, geneland assigned 86 individuals to the first cluster and 56 to the second one. Only two individuals were not assigned. Applying the same assignment threshold in T_2_, only seven individuals were not assigned as 158 individuals were assigned to another first cluster and 79 individuals to a second one. As expected under the admixture model, differences in membership profiles were found with tess (Appendix S2). In T_1_, tess assigned 30 and 70 individuals to the first and second parental populations, respectively, the other 44 individuals, that is ∼ 30% of the population, being admixed. In T_2_, the proportion of admixed individuals increased to ∼ 40% (i.e. 100 individuals). The remaining 93 and 51 individuals were assigned to two other parental populations.

**Figure 2:**
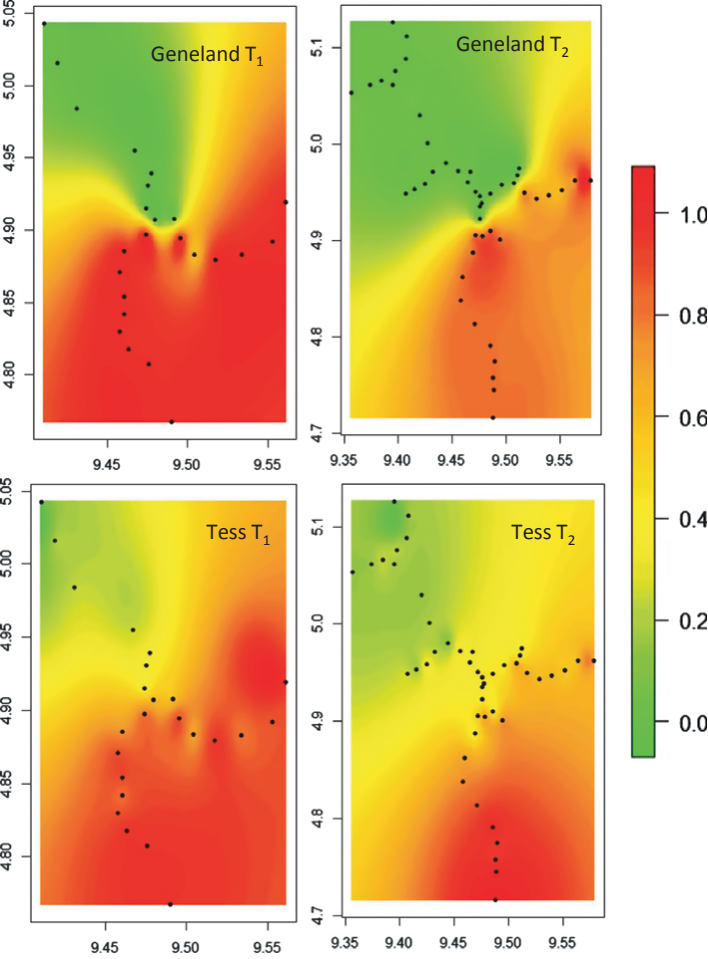
Spatial genetic structure of *Mycosphaerella fijiensis* populations over the study area. The maps either indicate the posterior probability of belonging to cluster 1 (geneland – upper panels) or to the first parental population (tess – lower panels) at both T_1_ and T_2_ sampling dates. Black dots represent sampled sites and *x-* and *y-*axes correspond to geographical coordinates.

The existence of a break in allelic frequencies was more conspicuous from geneland map but both algorithms exhibited consistent spatial genetic structures. Finally, the multilocus genetic discontinuity was located at approximately the same latitude (∼ 4.90°, Fig. 2) in both datasets.

Indices of genetic diversity for T_1_ and T_2_ samples are given in Appendix S3. Expected heterozygosity did not significantly vary between the two samplings (*U*-test *P* = 0.815). No pair of loci showed significant linkage disequilibrium after FDR control (*Q*-value < 0.05). Genetic differentiation (*F*_ST_) between the two clusters detected in geneland was significant in both samplings (*F*_ST_ = 0.091 at T_1_ and *F*_ST_ = 0.07 at T_2_) and greatly variable among microsatellite loci (Appendix S3).

### Cline description

**Figure 3:**
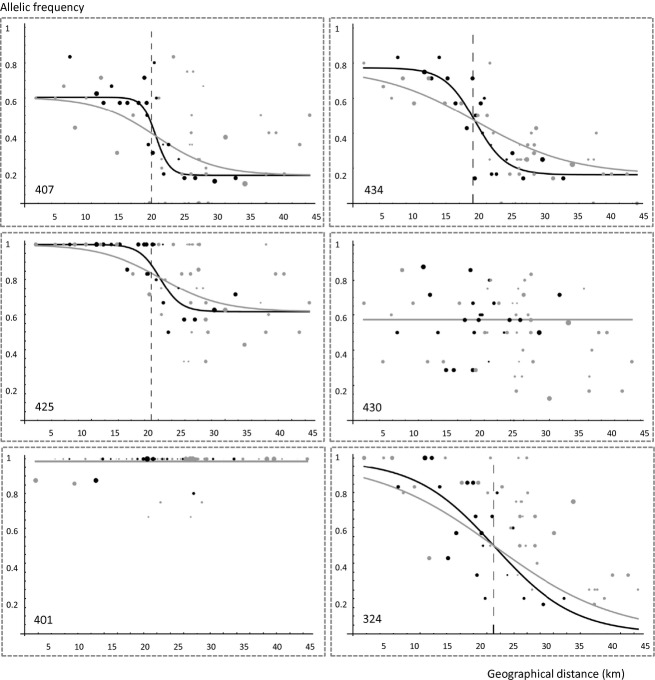
Change in neutral genetic clines for six of the 15 microsatellites considered between the two sampling dates. From top to bottom and left to right, the loci represented are MfSSR-407, 434, 425, 430, 401 and 324. Allelic frequencies at T_1_ (black) and T_2_ (grey) are plotted against distance along the main axis of the cline. Distances are given in km, 0 being the southernmost site. Dots represent sampling sites, dot size being proportional to the number of individuals sampled. Curves show the fitted clines (scaled Logit shapes), with their centres highlighted through dashed vertical lines.

Scaled Logit shape best fitted the data (Appendix S4). This is probably because for most loci, alleles are not fixed within parental populations (Fig. 3) Eight loci (of 15) presented a significant cline pattern (i.e. non-zero slope) (Table 1). Among these loci, cline widths at T_1_ ranged between 3 km and 39 km (Table 1), cline centres were coincident and cline widths always increased significantly between T_1_ and T_2_ (Appendix S5). Hence, these clines tended to vanish, as expected under the homogenising effect of gene flow. The seven remaining loci showed no cline pattern (zero-slopes) at both T_1_ and T_2_ (Appendix S5).

**Table 1:** Testing for an initial cline pattern in 15 microsatellites

MfSSR	407	425	405	322	324	413	434	340	401	430	362	417	428	203	350
LL_*T1*_ (k = 5)	−84.42	−46.74	−87.17	−83.71	−74.65	−85.89	−83.46	−82.19	−38.45	−16.41	−16.62	−88.39	−91.55	−75.26	−36.14
θ*	1.871	1.878	1.869	1.870	1.870	1.875	1.881	1.873	x	x	x	x	x	x	x
*c* (km)*	20.06	20.08	19.87	21.07	20.09	21.02	19.94	19.99	x	x	x	x	x	x	x
b*	68.33	140.19	11.20	25.73	17.25	35.72	38.97	22.92	0.10	0.03	0.07	0.09	0.12	0.02	0.06
*h*_1_*	0.13	0.62	0.00	0.08	0.02	0.16	0.17	0.00	0.67	0.99	0.96	0.45	0.46	0.12	0.85
h_p_*	0.59	0.94	0.95	0.64	0.98	0.69	0.78	0.70	0.01	1.00	1.00	0.05	0.00	0.10	0.04
*h*_2_†	0.64	0.98	0.95	0.67	0.98	0.74	0.82	0.70	0.67	1.00	1.00	0.47	0.46	0.21	0.85
Slope†	8.79	12.62	2.67	3.77	4.16	5.24	6.29	3.99	0.00	0.00	0.00	0.00	0.00	0.00	0.00
Width (km)†	6.44	3.14	39.30	17.10	25.51	12.32	11.29	19.20	x	x	x	x	x	x	x
LL_0_ Worse model (k = 1)	−95.54	−59.02	−93.36	−92.43	−84.69	−94.48	−96.29	−91.61	−38.46	−18.13	−18.13	−88.95	−93.19	−76.76	−37.14
Dev: −2LL (LL_0_–LL_*T1*_)	**22.24**	**24.55**	**12.40**	**17.44**	**20.07**	**17.17**	**25.65**	**18.84**	0.02	3.46	3.04	1.11	3.29	3.01	2.00

Clines were fitted using scaled Logit function (Equation in Appendix S6).

* Denotes inferred parameters: cline centre (*c*), cline slope-related parameter (*b*), lower asymptotic frequency (*h*_1_), allelic frequency step between the two populations (*h*_*p*_) and cline angle (θ).

† Holds for *a posteriori* computed parameters. These are *h*_2_, the higher asymptotic frequency 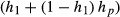, cline slopes 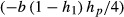 and for non-flat clines, cline widths (4/*b*, in km). LL is log likelihood. For each locus, slope significance was tested using likelihood ratio tests against a null model assuming homogeneous allele frequency over the study area. Deviance (Dev) is given with significant values (5% level) highlighted in bold × indicates situations in which parameters could not be computed (see text for details).

### Dispersal estimation

First, cline angle (θ = 1.87 radians ≈ 107°, support limit interval [1.64–1.98 radians]) was not significantly different between the two samplings (Table 2) and was congruent with the disposition of most sampling sites along the main road (Fig. 1). Second and importantly, the model with a single value of σ common to all loci was significantly better than the model with locus-specific values of *σ* (Table 2). The best model (model G) considers that centres are coincident between loci at T_1_ and that no cline movement has occurred (δ = 0) (Table 2). From the best model, σ was estimated at 1175 metres/generation^1/2^ with a support limit interval of [690 m–1586 m] (Appendix S7).

**Table 2:** Testing cline coincidence, similarity in cline vanishing rates between loci and isotropy. A. Compared models. Parameters constrained and type of constraint operated are highlighted in bold. σ is the standard deviation of parent–offspring axial dispersal distances, *b* a cline slope parameter, *h*_1_ lower asymptotic frequency, *h*_*p*_ the step in allelic frequency between the two populations, θ cline angle, *c* cline centre, and δ centre shift between T_1_ and T_2_. B. Model comparison on the whole dataset (both samplings and 15 loci). LL is log likelihood and *k* the number of parameters. Q_AIC_ corrects Akaike Information Criterion (AIC) for overdispersed frequency data and the best model is highlighted in bold

A	

Model	Parameters constrained
A	θT_1_≠θT_2_/*c*, *b*, *h*_1_, *h*_*p*_, δ & *σ*≠between locus
B	***θ*T_1_**=***θ*T_2_**/*c*, *b*, *h*_1_, *h*_*p*_, δ & *σ*≠between locus
C	***θ*T_1_**=***θ*T_2_**/*c*, *b*, *h*_1_, *h*_*p*_ & δ≠between locus/**σ=0**
D	***θ*T_1_**=***θ*T_2_**/*c*, *b*, *h*_1_, *h*_*p*_ & δ≠between locus/**s same σ for each locus**
E	***θ*T_1_**=***θ*T_2_**/*b*, *h*_1_, *h*_*p*_ & δ≠between locus/**same σ & c for each locus**
F	***θ*T_1_**=***θ*T_2_**/*b*, *h*_1_ & *h*_*p*_≠between locus/**same σ, c & δ for each locus**
G	***θ*T_1_**=***θ*T_2_**/*b*, *h*_1_ & *h*_*p*_≠between locus/**same σ & c for each locus/δ=0**
H	***θ*T_l_**=***θ*T_2_**/*c*, *b*, *h*_1_ & *h*_*p*_≠between locus/**same σ & δ for each locus**
I	***θ*T_l_**=***θ*T_2_**/*c*, *b*, *h*_1_ & *h*_*p*_≠between locus/**same σ for each locus/δ=0**

B					

Model	LL	k	AIC	Q_AIC_	AIC weight
**G**	−**2637.63**	**34**	**5343.26**	**3731.72**	**0.88**
F	−2638.68	35	5347.35	3735.17	0.11
I	−2636.09	41	5354.18	3743.58	0.00
D	−2635.17	49	5368.33	3758.30	0.00
C	−2638.93	48	5373.86	3761.53	0.00
B	−2627.74	63	5381.48	3775.98	0.00
E	−2649.25	42	5382.51	3763.87	0.00
A	−2627.29	64	5382.57	3777.35	0.00
H	−2652.63	42	5389.26	3768.56	0.00

### Influence of kernel on dispersal estimation

**Figure 4:**
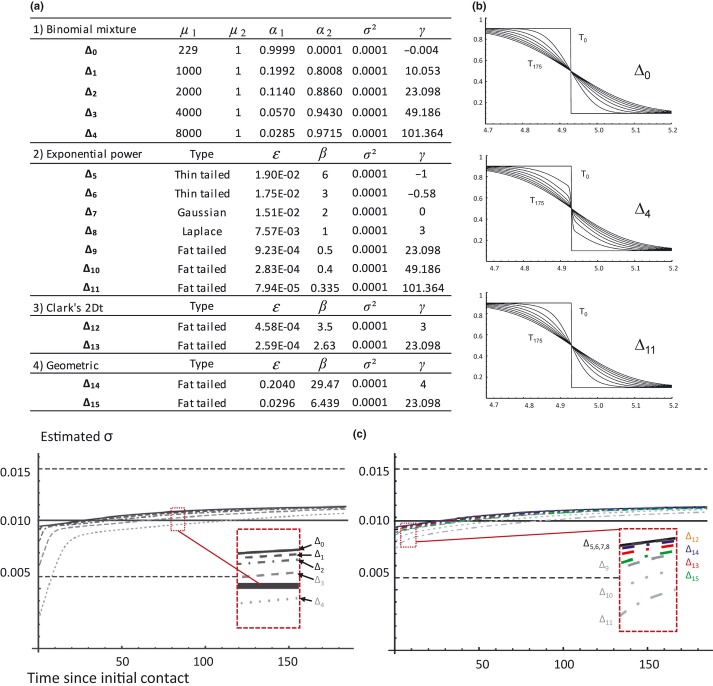
Influence of kernel on σ estimation. (a) Construction of dispersal distributions Δ_*i*_ using four different kernel families displaying identical variance (σ^2^, in degree^2^/generation) but different kurtosis (γ). μ, *α*, ε and β are kernel parameters (Appendix S9 for equations). (b) Simulated clines obtained from Δ_0_, Δ_4_, Δ_11_. Clines are shown every 25 generations from T = 0 to 175 generations after initial contact. (c) Temporal dynamics of σ estimate from simulated data sets. Left panel: mixture of Binomial distributions. Right panel: other dispersal kernels. Horizontal black solid line indicates the expected value (σ = 1175 m/generation^1/2^), and horizontal dashed lines its associated support limits.

Figure 4c shows the effects of the family and kurtosis of underlying dispersal kernels as well as time of first sampling on σ estimation. All estimates felt within the support limits obtained on the real dataset, indicating that the departure from diffusion approximation causes biases well below measurement error in our case. Only the kernel Δ_4_, a mixture of Binomial distributions with extremely high kurtosis, combined with a very early sampling date (T_1_ < 8 generations) could generate a detectable bias (underestimation of σ). Under these conditions, clines showed non-logit shapes with discontinuities in allele frequencies (Fig. 4b). Interestingly, an exponential power distribution with the same kurtosis value induced smaller biases (kernel Δ_11_). This suggests that moments of order higher than four (distribution tail) might be at the origin of the observed deviation with Δ_4_.

## Discussion

This study represents the first use of the decay of neutral clines to quantitatively infer dispersal. This approach basically requires genotyping individuals with neutral markers in a situation of secondary contact between differentiated populations in which drift can be considered as negligible in front of migration. It provided fairly accurate measure of contemporary gene flow and was robust to deviations from diffusion approximation.

### Estimation of dispersal in *Mycosphaerella fijiensis*

We provided the first estimate of dispersal in the fungal banana pathogen *M. fijiensis*. It is also among the first quantitative genetic-based estimates of dispersal at a landscape scale in fungal plant pathogens, a group that comprises most emerging infectious plant diseases (Fisher *et al*. 2012), and which ecology is strikingly poorly known. Up to now, population genetics studies have seldom allowed estimating dispersal because pathogen populations are frequently out of genetic equilibrium and/or suspected to have too high effective sizes (Giraud *et al*. 2008; Rieux *et al*. 2011). Most dispersal estimates have therefore relied on spore traps or field epidemiological studies often at very small geographical scales (McCartney *et al*. 2006). Here, we estimate σ, a synthetic diffusion parameter in which gene flow is averaged over 15 generations, which is also equivalent to the standard deviation of parent–offspring axial dispersal distances. Its value (σ = 1175 m/generation^1/2^) suggests very high dispersal abilities in *M. fijiensis*. Despite the absence of previous dispersal estimates in this species, this estimate is consistent with the biology of the fungus. *Mycosphaerella fijiensis* exhibits two natural modes of dispersal. The first relies on asexual spores (conidia), suspected to disperse at very short distances through rain splash (Gauhl 1994). The second is ensured by light sexual spores (ascospores) actively projected in the air (Gauhl 1994) and wind dispersed (Burt 2003). By analogy to other airborne fungi, *M. fijiensis* ascospores have been suspected to occasionally travel over several kilometres (Burt 2003; Amil *et al*. 2007). Finally, in addition to natural spore dispersal, the disease also spreads through human-mediated transport of infected plant material (Carlier 2000). This propagation mode may occur in the studied valley, as trades of banana plants between villages are common local agricultural practices. So important dispersal combined with high population sizes are also consistent with the recent non-detection of any Isolation By Distance (IBD) pattern along a 33-Km long transect (Rieux *et al*. 2011). The fact that no movement of cline centres was detected suggests isotropic dispersal over the study area (Barton & Hewitt 1985). Wind data are unavailable, but this result suggests either isotropic wind influences and/or isotropic movement of infected plants between villages surrounding cline centre over the last 2 years.

### Validity of the ‘pure-migration’ assumption

The present analysis relies on the existence of clines vanishing under the action of migration alone (i.e. without any selection or genetic drift). Both cline theory and empirical data have shown that linkage disequilibrium can build up easily in clines maintained by selection (Barton 1983). When this happens, any neutral locus gets more or less linked to loci under selection. Studying (neutral) microsatellite markers therefore does not guarantee that observed clines are not indirectly affected by selection or drift. Here, we had *a priori* reasons to argue that the observed genetic clines are not influenced by selection and drift. First, recent spore countings in Cameroonian populations revealed huge census population sizes probably high enough to avoid genetic drift (Rieux *et al*. 2012). Second, the sampled area is relatively small and characterised by an apparent absence of environmental or exogenous selective gradient (only one host species and no fungicide pressure). Third, contrary to hybrid zones in which distinct diploid races or subspecies meet, all sampled individuals are haploid and belong to the same species, which should limit the occurrence of endogenous selection against hybrids. Beyond *a priori* clues, one major advantage of this method is to provide tools to test for departures from the pure-migration hypothesis through a maximum likelihood framework. Here, the analysis did not reveal any impact of indirect selection. Clines were sigmoid rather than stepped as expected under strong indirect selection. The eight clines identified at T_1_ were all shown to significantly fade 15 generations later, suggesting that the genetic discontinuity is not at migration–selection equilibrium. Importantly, no heterogeneity in the rate of increase in cline width was detected among loci. If physically linked to a selected locus, neutral loci should see their cline width increase at a rate determined by the strength of selection against introgression at the selected locus (Barton & Gale 1993). Selection is therefore expected to result in contrasted rates of cline width increase at different microsatellites, which was not observed. Finally, the seven loci that showed no initial cline pattern behaved consistently with the pure-migration scenario, as no discordant geographical pattern appeared 15 generations later. Drift also appeared negligible here. Cline fits were of good quality as attested by low variance inflation factors (Table 2). Besides, if influenced by genetic drift, neutral loci should see the variance in allele frequency increase at random rates, leading again to heterogeneity in rates of cline width increase among loci, which was not observed.

### Robustness of the method

As most previous cline studies, the present approach relies on the underlying assumption that dispersal is a pure diffusion process (but see Saccheri *et al*. 2008 for an exception). Diffusion approximately corresponds with a Gaussian dispersal kernel. However, for many organisms, and especially wind-dispersed species such as *M. fijiensis*, dispersal has been shown to follow a leptokurtic kernel, that is with an excess of small- and long-distance dispersal in comparison to the Gaussian distribution (e.g. Sackett & Mundt 2005). Two previous studies have investigated the effects of varying kurtosis on clines at migration–selection equilibrium (Lenormand 1998; Rousset 2001). In both cases, it has been shown that large kurtoses may significantly increase cline maximum slope in comparison to a Gaussian dispersal distribution, leading to an underestimation of *σ*. As the present approach relies on a temporal comparison of cline slopes, it was not clear whether leptokurtic dispersal distributions would deteriorate dispersal estimation. Our simulations showed that the method is actually robust to leptokurtic kernels, as most estimated values remained within the study support limits. Results were very congruent among kernel families, showing that other aspects of dispersal distributions (e.g. tail) have low impact on the estimation. The only noticeable deviations were detected for Binomial mixture distributions, with exceptionally strong kurtosis (γ > 100) and only for early sampling dates. Such high kurtoses have never been reported from experimental studies (Appendix S8) and were only studied for illustration purposes. In this situation, clines present discontinuities in allele frequencies due to an excess of long-distance dispersal and a lack of short-distance mixing. Clines are then poorly fitted using smooth shapes and Endler∼s formula should not apply. Should the case arise with real data, such signature could be readily detected from the allele frequency pattern.

### Desirable species, markers and sampling

The method is formulated in a very general manner potentially applicable to a variety of organisms. One important prerequisite is that effective sizes be high enough for drift to be negligible in front of migration in studied populations. This clearly disqualifies the method for species with very low effective sizes. For these, one may count on other methods, such as mark-recapture methods, to measure dispersal. Our approach may nevertheless represent a powerful alternative for various species characterised by moderate to high effective sizes. Importantly, several of its key assumptions (including drift effect) can be internally checked, so that the method should overall provide robust estimates.

Using bi-allelic markers is not a prerequisite, as robust approaches exist to convert multi-allelic markers into a bi-allelic system (see for instance Daguin *et al*. (2001) and references therein). Nevertheless, genetic markers displaying a small number of alleles should be preferred to avoid introducing additional variance in cline fits. We would thus recommend considering long tandem repeats markers or ideally single-nucleotide polymorphism markers (SNPs).

One other critical aspect of the method is sampling design. First, identifying a local contact zone clear enough so that individuals can be assigned without ambiguity to differentiated clusters is not required, as illustrated here with tess results. Importantly, sampling should (1) encompass individuals at both extremities of the contact zone so that cline plateaux can be properly estimated and (2) be intensified near the contact zone for cline centre and slope to be accurately inferred. If more than two populations meet, similar sampling advices apply as the method would allow taking advantage of such design by simultaneously fitting clines from several locations. Second, if dispersal truly occurs in 2D, sampling should also be performed in 2D, for cline main axis to be correctly inferred. Such precaution is necessary because if sampling is designed as a 1D transect along an inappropriate direction that forms an angle θ with cline main axis, inferred cline slopes will be multiplied by a factor Cos(*θ* ), resulting in overestimating *σ* by a factor 1/|Cos(*θ* )|. Here, secondary contact occurred in a shallow valley, where pathogen host plants are continuously distributed along roadsides. The valley is not a straight line and secondary axes exist (Fig. 1), so that we considered unsafe to making an *a priori* guess on cline angle and used a 2D approach. Cline angle was weak relative to the direction of the main road with a narrow support limit, which confirms that dispersal actually mainly occurs along roadsides. In this specific situation, a 1D approach would have resulted in a relatively minor error on dispersal estimate.

### Back to the historical contact time

In principle, from Endler∼s model, one may estimate the product 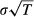, where *T* is the time since historical contact in generations (eqn 3). Using two samplings, we ideally would have been in a position to jointly infer σ and *T*. We would have written the likelihood of T_1_ clines as a function of both parameters. This was not possible here for one reason. Under pure migration, clines formed subsequently to a secondary contact should have the same width across loci. This was not true in our data as cline widths at T_1_ differed. This variability in initial cline widths suggests a major role of drift during the initial stages of cline formation and is consistent with the historical expansion of *M. fijiensis* in Cameroon. This species is self-incompatible: the production of sexual spores requires high enough population size for two sexually compatible lesions to germinate, grow and collapse on the same leaf. This Allee effect may have constrained initial population spread to rely on asexual spores, possibly leading to an increased intensity of drift. Such limitation may not hold for other species, for which the method would allow estimating contact time.

## Conclusion

Our results show that combining genetic clustering approaches to detect breaks in allelic frequencies with neutral genetic cline analyses offers a convenient way to gain solid information on the rate of contemporary dispersal. It combines the desirable properties of both direct methods (e.g. estimation over a known and recent period) and classical indirect approaches (e.g. measure of effective dispersal). With the continuous development of both new sequencing technologies and spatial genetic clustering algorithms (Guillot *et al*. 2009), we expect that opportunities to detect such fine-scale spatial genetic discontinuities will multiply in the future. The neutral cline approach may thus become a useful and cost-effective method to infer dispersal, particularly in emerging or invasive species. By applying it to the fungal pathogen, *M. fijiensis*, we have provided evidence for the high dispersal abilities of this species. Obtaining landscape-scale estimates of contemporary dispersal in a wider range of species is now crucial for ecological and evolutionary principles to guide the design of quarantine and management policies (e.g. rationalisation of pesticide use, deployment of resistant varieties).
