# Supplementary material for: Using neutral cline decay to estimate contemporary dispersal: a generic tool and its application to a major crop pathogen
Source: Ecol Lett. 2014 Jul 22;16(6):721–30. doi: 10.1111/ele.12090 (PMC4165305; doi:10.1111/ele.12090)
Supplement: Supplementary file 1 [file ele0016-0721-SD1.doc]

**Appendices to manuscript entitled:**

**Using neutral cline decay to estimate contemporary dispersal: a generic tool and its application to a major crop pathogen**

A. RIEUX ^*’^, T. LENORMAND **^¶^**, J. CARLIER ^*^, L. DE LAPEYRE DE BELLAIRE ^‡^ and V. RAVIGNE ^*^

Appendix S1: Correspondence between sampling sites in Rieux et al. (2011) and those considered in the present study.

Appendix S2: Inference of the number of cluster(s) (k) and assignment probabilities using GENELAND and TESS.

Appendix S3: Summary of basic genetic diversity results.

Appendix S4: Finding the best cline shape.

Appendix S5: Likelihood ratio tests for locus/locus vanishing cline patterns test.

Appendix S6: Details on the model used for estimation of σ.

Appendix S7: Likelihood profile for the estimation of σ.

Appendix S8: Examples of kurtosis values found in the literature.

Appendix S9: Dispersal kernel families investigated using simulations.

References cited

Appendix S1: Correspondence between sampling sites in Rieux et al. (2011) and those considered in the present study.

• Rieux et al. (2011) sampling sites

Area including the sites considered in the present study


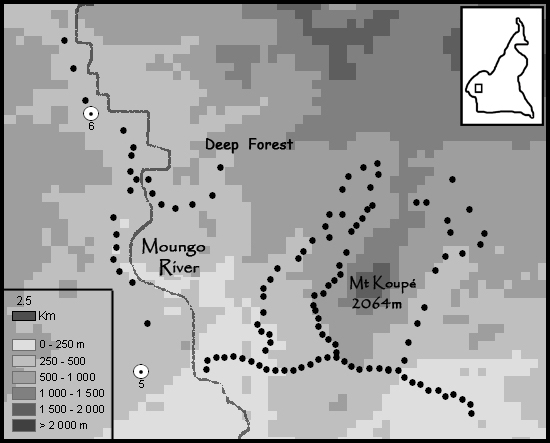


Figure S1. Graphical representation of the sampling sites from Rieux et al. (2011) that have been also considered in the present study (T_1_ sampling).

Appendix S2: Inference of the number of cluster(s) (K) and assignment probabilities using GENELAND and TESS.

|  |  |  |  |  |
| --- | --- | --- | --- | --- |
| K | GENELAND T_1_ | GENELAND T_2_ | TESS T_1_ | TESS T_2_ |
| *1* | 6.31 (4.81) | 2.16 (1.03) | - | - |
| *2* | **69.26 (3.46)** | **61.21 (5.72)** | **2097.13 (7.45)** | **3687.68 (6.22)** |
| *3* | 15.41 (1.29) | 28.33 (2.75) | 2152.54 (12.07) | 3703.48 (9.01) |
| *4* | 3.12 (1.87) | 4.56 (3.77) | 2251.29 (4.77) | 3733.62 (8.77) |
| *5* | 0.16 (0.93) | 1.35 (0.44) | 2410.38 (8.74) | 3819.09 (3.75) |
| *6* | 0.97 (1.63) | 2.09 (0.99) | 2382.94 (1.01) | 4053.82 (12.99) |
| *7* | 1.23 (1.04) | 0.03 (0.17) | 2389.06 (3.64) | 4156.39 (1.48 |
| *8* | 2.73 (0.98) | 0 (0) | 2508.51 (5.75) | 4038.44 (3.69) |
| *9* | 0 (0) | 0.27 (1.39) | 2592.95 (1.47) | 4216.53 (5.78) |
| *10* | 0.81 (0.57) | 0 (0) | 2442.72 (3.11) | 4202.76 (6.74) |
|  |  |  |  |  |

Table S2. Inference of the number of cluster(s) (*K*) that best explains observed spatial genetic structure. For Geneland we reported the percentage of iterations spend by the RJMCMC on each K across simulations after the burning period. For Tess we reported the values of DIC (Deviation Index Criterion), smallest values reflecting the best number of parental populations. It is noteworthy that it is not possible to run Tess for *K* = 1 (as a test of no population structure). Both indices were averaged over 5 runs with standard deviation indicated inside brackets. T_1_ holds for first sampling, and T_2_ for second sampling.

Assignment probability to the first cluster

Assignment probability to the first cluster


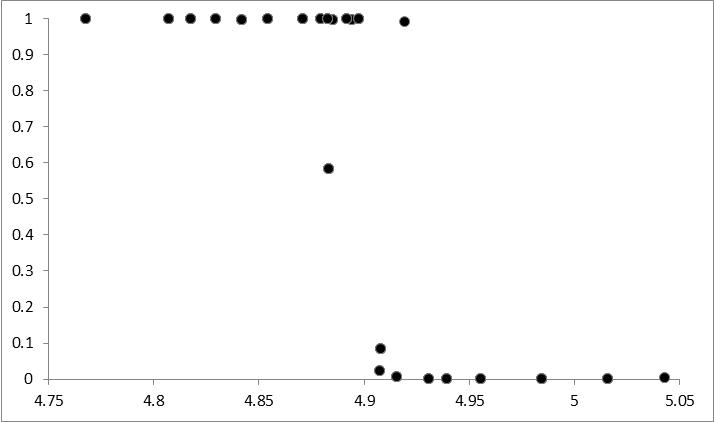

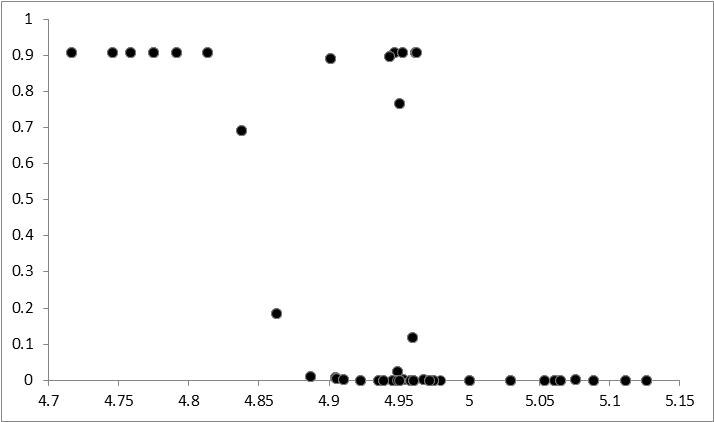


GENELAND T_1_

GENELAND T_2_

Latitude

Latitude


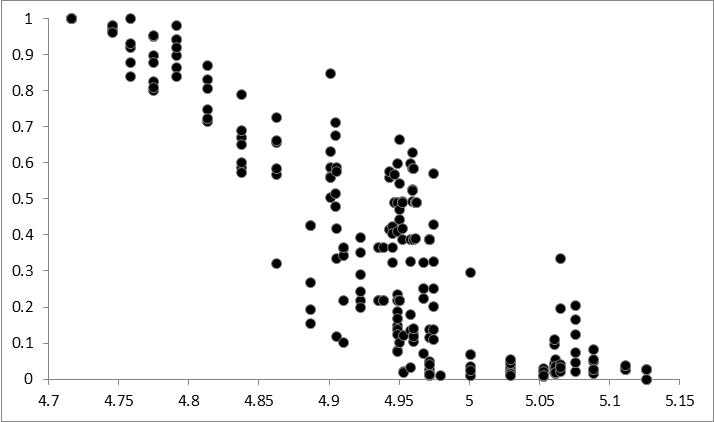

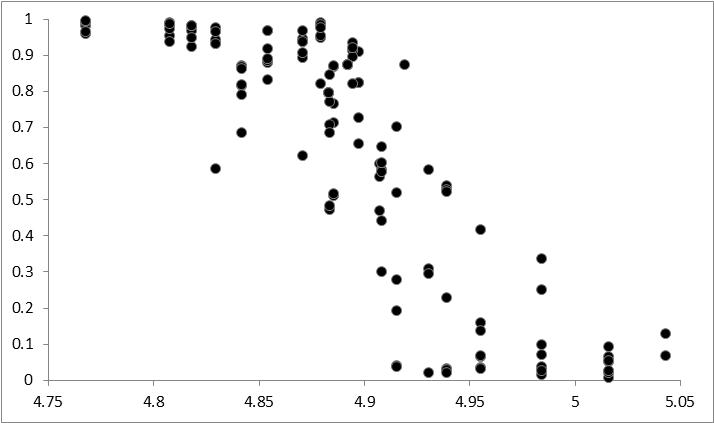


Assignment probability to the first parental population

Assignment probability to the first parental population

TESS T_1_

TESS T_2_

Latitude

Latitude

Figure S2. Assignment probability of either each individual to one of the two clusters (GENELAND) or each individual genome to one of the two parental populations (TESS) at both sampling times (T_1_ and T_2_). Each point represents an individual. The difference in number of points between GENELAND and TESS is due to the fact that GENELAND displays an average probability of assignment over individuals sampled at the same locality.

Appendix S3: Summary of basic genetic diversity results

Table S3. Summary of basic information and population genetic analyses of both temporal samplings (*T_1_* and *T_2_*) computed for all microsatellite loci. *N* is sample size. For each locus, we provide *H_E_*, the unbiased estimate of gene diversity, *Ar*, the number of alleles and the *F*_ST_ between the two GENELAND clusters with their significativity according to Fisher’s exact test (NS > 0.05; * P < 0.05; ** P < 0.01; *** P < 0.001). The number into brackets indicates the number of repeat unit nucleotides for each microsatellite locus.^‡^ Indicates the loci that were also at use in the previous study (Rieux et al. 2011). Nomenclature has changed between the two studies. MfSSR340, 362, 322, 324, 350 and 203 now respectively state for F40, F62, Fe05b, Fe09, FF09 and N203.

Appendix S4: Finding the best cline shape

Three different shapes were fitted to allelic count data.

• Shape A is called stepped cline and corresponds to a three-part function containing a central sigmoid and two exponential tails. Two parameters characterize the central sigmoid (center *c* and slope *b*) and four parameters characterize the two tails (distance from the centre at which the right and left exponential tails start and their relative slopes, *d*1, *d*2, *t*1 and *t*2 respectively). Such a shape is expected in tension zones involving strong indirect selection across the genome (Szymura and Barton 1986).

$$1-\frac{1}{1+e^{-bd_{2}}}e^{\left(\frac{-bt_{2}(x-c+d_{2})}{1+e^{bd_{2}}}\right)}$$

for *x* ≤ *c*- *d*_2_

for *c* - *d*_2_ ≤ *x* ≤ *c* + *d*_1_

S4.1

$$1-\frac{1}{1+e^{-bd_{1}}}e^{\left(\frac{-bt_{1}(x-c+d_{1})}{1+e^{bd_{1}}}\right)}$$

for *x* ≥ *c* - *d*_1_

• Shape B is a simple Logit (two parameters). In such a shape, up and down asymptotic frequencies (plateaux) are automatically fixed to a value of 1 and 0 respectively (Szymura and Barton 1986). It is expected when when populations with diagnostic alleles meet (Endler 1977).

$$\frac{e^{b(x-c)}}{1+e^{b(x-c)}}$$

S4.2

The logit shapes naturally result from the resolution of the diffusion equation with the assumption of a linear secondary contact at time 0. These initial conditions assume that the two populations meet along a straight line and that:

$\frac{\partial p(x,y,t)}{\partial t}=\frac{\sigma^{2}}{2}\nabla^{2}p(x,y,t)$S4.3

$p(x,y,0)=h_{1}$ for all *x*, *y* on a side of the contact line and

$p(x,y,0)=h_{2}==h_{1}+h_{p}\left(1-h_{1}\right)$ for all *x*, *y* one the other side of the contact line.

Note that the exact solution is in the form of an error function and that it is finely approximated using Logit fits.

• Shape C is a scaled Logit in which both up (h_2_) and down (h_1_) asymptotic frequencies might take any value between 0 and 1 (total of four parameters). It is expected in a contact zone with semi-diagnostic alleles (Endler 1977).

$h_{1}+\left(h_{2}-h_{1}\right)\frac{e^{b(x-c)}}{1+e^{b(x-c)}}$

S4.4

To determine the best description of the data, we started by fitting those three different shapes to multilocus genetic data (15 Loci) independently for each temporal sampling (*T*_1_ and *T*_2_) and without setting any constraint onto parameters. Note that because we considered 2D versions of these functions,

$x=X\cos\left(\theta\right)+Y\sin\left(\theta\right)$ S4.5

where *X* and *Y* are sample longitude and latitude and θ the angle that the cline axis forms with the parallel. As a consequence, one supplementary parameter should be added to each of these three shapes to fit θ, resulting in the existence of *k* = 7, 3 and 5 parameters for shape A, B and C respectively for each sampling date and locus (thus explaining the total numbers of parameters in table S4.

**
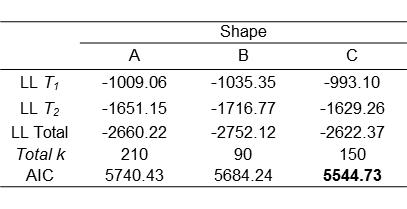
**

Table S4. Log likelihood (LL), total number of parameters (Total k) and corresponding *AIC* for the multilocus clines (15L) independently fitted for each sampling. Best value is highlighted in bold.

Appendix S5: Tests of a vanishing cline pattern for each of the 15 microsatellite markers

Table S5. Tests of a vanishing cline pattern for each of the 15 microsatellite markers (MfSSR). Each locus was fitted using the scaled Logit shape. For each locus, a vanishing cline pattern was tested using likelihood ratio tests (LRT). LRTs were realized between the sum of likelihood of the two independent unconstrained models (LL*_T1+T2_*= LL*_T1_*+ LL*_T2_*) with the likelihood of a model in which both the slope and the asymptotic frequencies were constrained to be the same between *T_1_* and *T_2_* (LL_0_). Significant values (5% level) are highlighted in bold (Dev holds for deviance). Numbers of parameters *k* are in brackets.

Appendix S6: Details on the model used for the estimation of σ

In the present paper, clines were appropriately modelled using a scaled logit shape. At date *i*, the allelic frequency along the cline is:

$p_{i}\left(x\right)=h_{1}+h_{p}\left(1-h_{1}\right)\frac{e^{b_{i}(c_{i}-x)}}{1+e^{b_{i}(c_{1}-x)}}$S6.1

where *x* is the position along the cline, *c_i_* is cline centre at date *i*, *b_i_* is a slope parameter (see below), *h*_1_ is the lower asymptotic frequency and *h_p_* is the step between the lower and upper asymptotic allelic frequencies. Cline higher asymptotic frequency *h*_2_ is parameterized as:

$h_{2}=h_{1}+h_{p}\left(1-h_{1}\right)$ S6.2

**Cline width of scaled logit clines**

Cline width is defined as the ratio of the maximal difference in allele frequencies along the cline and the maximal slope of the cline (i.e., slope at cline centre). So at date *i*, cline width is:

$w_{i}\equiv\frac{\left|p_{i}\left(+\infty\right)-p_{i}\left(-\infty\right)\right|}{p'_{i}\left(c\right)}$ S6.3

With the scaled logit shape,

$\left|p_{i}\left(+\infty\right)-p_{i}\left(-\infty\right)\right|=h_{p}\left(1-h_{1}\right)$ S6.4

and

$p'_{i}\left(c_{i}\right)=\frac{b_{i}}{4}h_{p}\left(1-h_{1}\right)$S6.5

Consequently, cline width is simply:

$w_{i}=\frac{4}{b_{i}}$ S6.6

**Relationship between the change in cline shape and *σ***

From Endler’s (1977) relationship:

$w_{i}=\sigma\sqrt{2\pi T_{i}}$ S6.7

This translates into:

$T_{i}=\frac{w_{i}^{2}}{2\pi\sigma^{2}}$ S6.8

Therefore the number of generations between the two sampling events is:

$T=T_{2}-T_{1}=\frac{w_{2}^{2}-w_{1}^{2}}{2\pi\sigma^{2}}$ S6.9

Using Equation S6.6, this becomes:

$T=\frac{8}{\pi\sigma^{2}}\left(\frac{1}{b_{2}^{2}}-\frac{1}{b_{1}^{2}}\right)$ S6.10

Solving for *b_2_*, we get:

$b_{2}=\frac{1}{\sqrt{\frac{1}{b_{1}^{2}}+\frac{\pi T\sigma^{2}}{8}}}$ S6.11

It is further considered that cline center moves by a step *δ*, such that:

$c_{2}=c_{1}+\delta$ S6.12

At date *T_2_*, the allelic frequency along the cline is thus:

$$p\left(x\right)=h_{1}+h_{p}\left(1-h_{1}\right)\frac{e^{b_{2}(c_{1}+\delta-x)}}{1+e^{b_{2}(c_{1}+\delta-x)}}$$

S6.13

**What the modified version of CFIT does**

For each locus in the dataset, CFIT fits equation S6.1 to the first sampling event and equations S6.11 and S6.13 to the second one using maximum likelihood. Such a parameterization allows obtaining locus-specific estimates of *σ* and testing hypotheses by constraining some parameters (*c*_1_, *b*_1_, *δ*, *σ*) between loci or sampling events to be the same. In its current version, Cfit only allows estimating dispersal considering the scaled logit shape but it could be easily adapted to any other function.

Appendix S7: Likelihood profile for the estimation of σ

Figure S7. Likelihood profile for *σ* (in m/generation^1/2^). Dashed arrow indicates the 95% support limit interval around the likelihood maximum (black point) obtained from model G (see Table 2 for details on the different models).

Appendix S8: Examples of kurtosis values found in the literature

$\frac{\mu_{4}}{\sigma^{4}}-3$Table S8. Examples of normalized kurtosis values found in the literature. The (*) indicates that we did not explicitly find the kurtosis value in the paper of reference but had to compute it from parameter values indicated in the study as γ = **.**

In this equation, *µ*_4_ and *σ*_2_ are respectively the fourth and the second moment around the mean.

Appendix S9: Dispersal kernel families investigated using simulations.

In addition to the use of the mixture of Binomial (equation can be found in Lenormand 1998), we assessed the robustness of the estimation of *σ* using three other families of functions commonly used to model dispersal kernels. These are the, Exponential power, Clark’s 2Dt and Geometrical distribution families (equations can be found in Klein et al. 2006). Equations from Klein et al. 2006 were modified such as the scale parameter sums at 1 (because of the 1D nature of our simulations). *Γ* stands for the gamma function such as:

($\Gamma\left(x\right)=\int_{0}^{+\infty} t^{x-1}e^{-t}dt$) S9.1

**Mixture of Binomial distribution family**

$m\left(x\right)=\alpha_{1}B(2\mu_{1},\frac{1}{2})+\alpha_{2}B(2\mu_{2},\frac{1}{2})$ S9.2

with *B* holding for Binomial distribution

**Exponential power family:**

$m\left(x\right)=\frac{\beta}{2\varepsilon\Gamma\left(1/\beta\right)}\exp\left(-\left(\frac{\left|x\right|}{\varepsilon}\right)^{\beta}\right)$ S9.3

with *β* > 0, *ε* > 0

**Geometric family:**

$m\left(x\right)=\frac{\beta-1}{2\varepsilon}\left(1+\frac{\left|x\right|}{\varepsilon}\right)^{-\beta}$ S9.4

with *β* > 5 and *ε* >0.

**Clark’s 2Dt family:**

$m\left(x\right)=\frac{\Gamma\left(\beta\right)}{\sqrt{\varepsilon\pi}\Gamma\left(\beta-\frac{1}{2}\right)}\left(1+\frac{x^{2}}{\varepsilon}\right)^{-\beta}$ S9.5

with *β* > 5/2 and *ε* >0.

References cited:

Austerlitz F., Dick C.W., Dutech C., Klein E.K., Oddou-Muratorio S., Smouse P.E. & Sork V.L. (2004). Using genetic markers to estimate the pollen dispersal curve. *Mol. Ecol.*, 13, 937-954.

Barton N.H. & Hewitt G.M. (1982). A measurement of dispersal in the grasshopper *Podisma pedestris* (Orthoptera, acrididae). *Heredity*, 48, 237-249.

Endler J.A. (1977). Geographic variation, speciation, and clines. *Monographs in Population Biology*, 10, 1-246.

Klein E.K., Lavigne C., Picault H., Renard M. & Gouyon P.H. (2006). Pollen dispersal of oilseed rape: estimation of the dispersal function and effects of field dimension. *Journal of Applied Ecology*, 43, 141-151.

Lenormand T. (1998). Dynamique de l'adaptation locale: Résistance aux insecticides chez *Culex pipiens*. In:. PhD thesis Ecole Nationale Supérieure Agronomique de Montpellier

Sackett K.E. & Mundt C.C. (2005). The effects of dispersal gradient and pathogen life cycle components on epidemic velocity in computer simulations. *Phytopathology*, 95, 992-1000.

Szymura J.M. & Barton N.H. (1986). Genetic-analysis of a hybrid zone between the fire-bellied toads, *Bombina-bombina* and *Bombina-variegata*, near cracow in southern poland. *Evolution*, 40, 1141-1159.

Waser P.M. & Elliott L.F. (1991). Dispersal and genetic-structure in kangaroo rats. *Evolution*, 45, 935-943.
